# In silico structural and functional characterisation of two hypothetical proteins from the plant‑associated bacterium *Kosakonia cowanii* F52

**DOI:** 10.1186/s44342-026-00080-9

**Published:** 2026-09-30

**Authors:** Md Zahirul Islam, Md Jahid Hasan Dewan, Abdul Baess Keyhani, Monira Fayaz

**Affiliations:** 1 College of Life Sciences, Northwest A&F University, Yangling, Shaanxi 712100 China; 2 Department of Microbiology, Jagannath University, Dhaka, 1100 Bangladesh; 3 Hubei Hongshan Laboratory, Hubei Engineering Technology Research Centre for Forestry Information, College of Horticulture and Forestry Sciences, Huazhong Agricultural University, Wuhan, 430070 China; 4 College of Plant Sciences and Technology, Huazhong Agricultural University, Wuhan, 430070 China

**Keywords:** *Kosakonia cowanii*, Hypothetical proteins, AlphaFold2, Rossmann fold, ARM-repeat, Proline biosynthesis, Salt tolerance

## Abstract

*Kosakonia cowanii* promotes plant growth under salt stress, but many of its hypothetical proteins (HPs) remain uncharacterised. We screened 218 HPs from *K. cowanii* F52 using sequential filters for physicochemical stability, subcellular localisation, and conserved domains. Four candidates were modelled with AlphaFold2 and validated by stereochemical and global geometry scores. Two HPs, MHQ1860809.1 and MHQ1862734.1, were selected based on combined global and per-residue model-quality metrics for further functional annotation via structural similarity searches, Gene Ontology (GO), and KEGG pathway analysis. Molecular docking of NAD⁺ was subsequently performed on MHQ1860809.1 to evaluate cofactor binding. MHQ1860809.1 is predicted to adopt a canonical Rossmann fold with a conserved NAD(P)-binding pocket; docking was consistent with NAD⁺ binding (Vina score −7.8 kcal/mol). GO predicted oxidoreductase activity (GO:0016646) and proline metabolic process (GO:0006560). MHQ1862734.1 is predicted to fold into an ARM-repeat superhelix, lacks any ligand-binding cavity, and is structurally similar to importin-β and AP-2 adaptor subunits. However, no EC numbers or KEGG orthologies could be assigned. MHQ1860809.1 is therefore structurally consistent with a putative NAD(P)-dependent oxidoreductase potentially involved in proline biosynthesis, whereas MHQ1862734.1 adopts an ARM-repeat fold of unknown function. These findings generate specific, testable hypotheses and provide a foundation for future experimental work on the molecular basis of K. cowanii plant growth-promoting activity.

## Introduction

Plant-associated bacteria are gaining recognition as key contributors to sustainable agriculture. These microbes provide biological substitutes for synthetic fertilizers and pesticides through strategies such as phytohormone synthesis, phosphate solubilisation, iron chelation via siderophores, and ACC deaminase-mediated ethylene reduction [1, 2]. Among these, *Kosakonia cowanii* (*K. cowanii*) is a Gram-negative member of the *Enterobacteriaceae* and has emerged as a multifunctional plant growth-promoting rhizobacterium (PGPR) [3]. It is documented to enhance crop salinity tolerance, suppress fungal pathogens, solubilise phosphate, and produce phytohormones, serving as both a biostimulant and a biofertilizer [4, 5]. Unlike model PGPR such as *Pseudomonas* or *Bacillus*, *K. cowanii* lacks a dedicated curated molecular database, and its beneficial traits have been described almost exclusively at the physiological level. Most studies have reported increases in biomass or disease reduction without identifying the proteins or molecular mechanisms responsible, a knowledge gap that hinders the rational design of robust bioinoculants to mitigate the impact of soil salinisation on crop yields.

The plant growth-promoting potential of *K. cowanii* is exemplified by the fully sequenced strain F52 (GenBank CM160069.1), whose genome provides an opportunity to probe its molecular machinery. A major obstacle to decoding PGPR activity is the prevalence of hypothetical proteins (HPs), sequences with no experimentally or computationally assigned function [6]. In this genome, 5.1% of the proteins are annotated as HPs. These uncharacterized sequences could harbour novel enzymes, signaling adaptors, or effector proteins that directly mediate plant growth promotion and stress tolerance [7]. However, traditional homology-based tools such as BLAST fail for most HPs because they lack detectable sequence similarity to experimentally validated entries, leaving them in a permanent functional blind spot [8]. Consequently, the very proteins that might be most unique to *K. cowanii* F52, and most responsible for its halotolerant PGPR traits, remain invisible to conventional annotation pipelines.

Protein three-dimensional structure is far more evolutionarily conserved than amino acid sequence, a principle that allows functional inference even when sequence similarity is undetectable. Many proteins retain their overall folds across kingdoms despite sequence identities often falling below 15%, a phenomenon known as the “structure outlives sequence” paradigm [9]. This means that if we can predict the structure of a hypothetical protein with high confidence, we can often infer its function by comparing that structure to experimentally determined ones, even in the absence of detectable sequence homology [10, 11]. The recent revolution in deep learning-based structure prediction, led by AlphaFold2, now delivers near-experimental accuracy for single-domain proteins, making structure-guided annotation feasible at scale [12, 13]. This approach allows us to systematically probe the “functional dark matter” of a PGPR genome without the months or years required for experimental structure determination [14]. As a result, computational annotation of hypothetical proteins has shifted from a speculative exercise to a rigorous, high-throughput strategy that generates testable hypotheses for experimental validation.

Building on this principle, we applied a computational pipeline to systematically screen and annotate hypothetical proteins from *K. cowanii* F52. Using sequential filters for stability, subcellular localisation, and conserved domains, we reduced the total HPs to four candidates for de novo structure prediction with AlphaFold2. After model validation, the two highest-quality models were selected for in-depth functional annotation through structural similarity searches, Gene Ontology and enzyme prediction, KEGG pathway mapping, and molecular docking of a representative cofactor. This multi-layered approach uses only publicly available data and established tools. This study does not introduce a new algorithm or pipeline; rather, it applies well-established structure-prediction and annotation tools to a genome for which no curated functional database exists. The output of this study is therefore a set of computational hypotheses for experimental validation, not validated functional assignments.

## Materials and methods

### Sequence retrieval and stability filtering

The complete genome of *Kosakonia cowanii strain* F52 (GenBank accession number: CM160069.1) was retrieved from the National Center for Biotechnology Information (NCBI). According to the database record, the strain was originally isolated from the wild fig tree *Ficus citrifolia* in Larry and Penny Thompson Memorial Park, Florida, USA, collected in August 2025. The genome is 4,708,407 bp in size with 4261 protein-coding sequences. Of these protein-coding sequences, 218 were annotated as hypothetical proteins (HPs) and retrieved in FASTA format. Their physicochemical properties were evaluated using the ExPASy ProtParam tool [15]. Proteins with an instability index greater than 40 were considered unstable and discarded, leaving 111 stable HPs.

### Subcellular localisation filtering

Subcellular localisation was predicted for the 111 stable HPs using four independent computational tools: CELLO v2.5 [16], PSORTb [17], SOSUIGramN [18], and PSLpred [19]. Only those HPs for which all four tools returned identical predictions were retained, resulting in 11 candidates.

### Conserved domain filtering

Conserved domains were searched in the 11 candidates using two resources: the NCBI Conserved Domain Database batch search and InterProScan [20]. InterProScan integrates multiple databases, including Pfam, PROSITE patterns, SUPERFAMILY, and Gene3D [21]. A hit was considered significant if its *E*-value was below 0.01. After removing seven proteins that had no significant domain, four candidates remained: MHQ1859537.1, MHQ1860643.1, MHQ1860809.1, and MHQ1862734.1.

### *Homology modelling attempt and *de novo* structure prediction*

The four candidate sequences were submitted individually to the SWISS-MODEL server for template-based homology modelling [22]. Although experimental templates were identified for all 4 proteins, the highest sequence identity to an experimentally determined structure (X-ray or cryo-EM) ranged from 14.1% to 21.2%, far below the 80% threshold required for reliable homology modelling. Therefore, de novo prediction was performed using AlphaFold2 [11], through the ColabFold environment [23]. The predictions were run without external templates (template_mode = none), and AMBER relaxation (num_relax = 5) was applied to the top-ranked model. AMBER relaxation performs energy minimisation prior to quality assessment. A single relaxed PDB file was retained for each of the four candidates.

### Model quality assessment and candidate selection

The relaxed AlphaFold2 models were validated using four standard quality-assessment tools. PROCHECK [24] generated Ramachandran plot statistics, from which the percentage of residues in the most favoured regions was recorded. ERRAT [25] provided an overall quality factor based on non-bonded atomic interactions. Verify3D [26] assessed the compatibility of the 3D model with its own amino acid sequence, and the percentage of residues with a 3D-1D score ≥ 0.1 was noted. QMEANDisCo [27] gave a global quality score and a QMEAN4 Z-score.

**Fig. 1:**
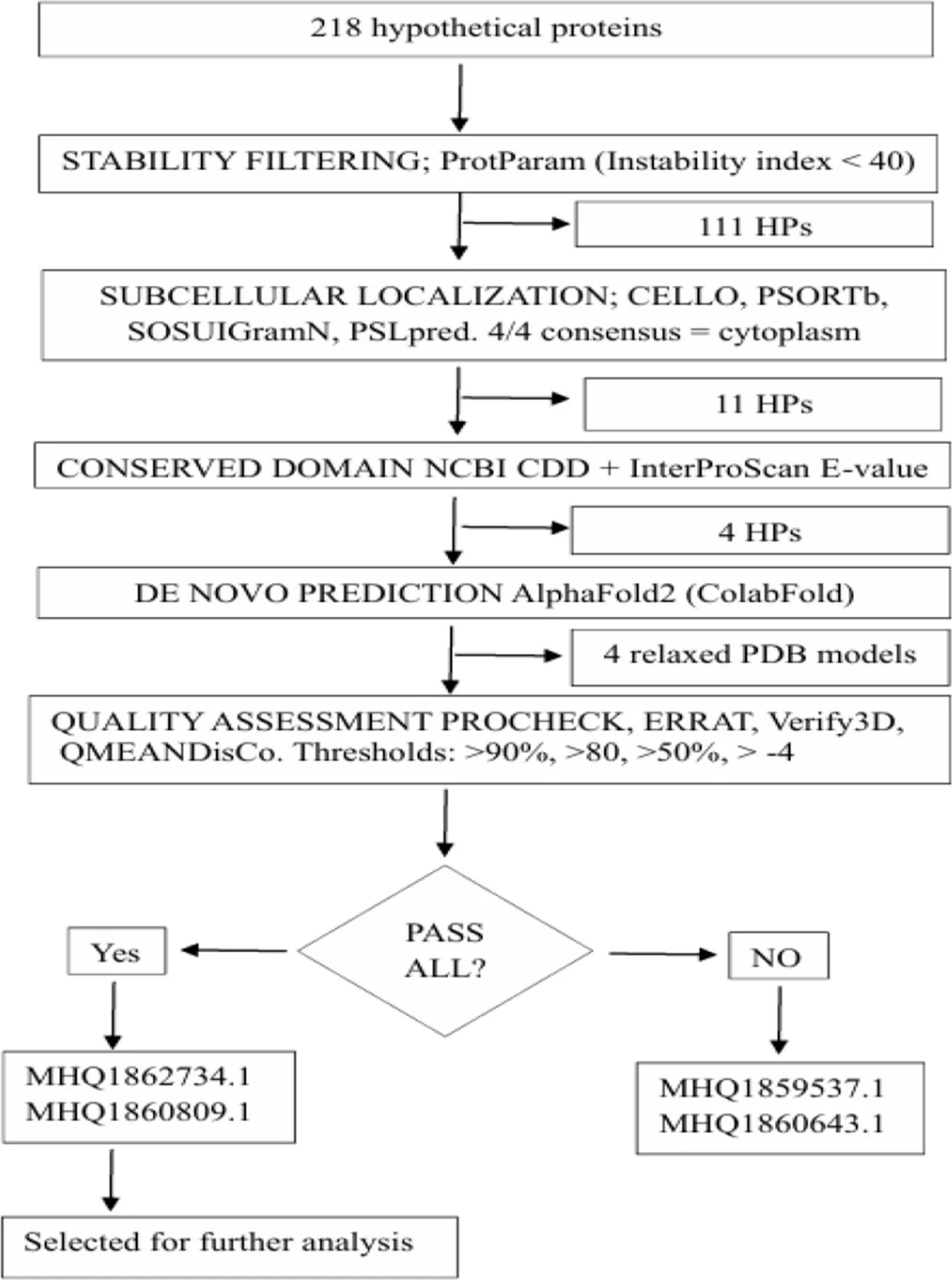
Workflow diagram illustrating the stepwise computational pipeline used to screen and select hypothetical proteins from *Kosakonia cowanii* F52

The following acceptance thresholds were applied: PROCHECK > 90% most favoured, ERRAT > 80, Verify3D > 50% at the 0.1 level, and QMEAN *Z*-score > −4. Based on the combined validation metrics, MHQ1862734.1 passed all thresholds (PROCHECK 91.6%, ERRAT 100, Verify3D 52.17%, QMEAN *Z*-score −0.29). MHQ1860809.1 passed PROCHECK (92.6%) and QMEAN (*Z*-score −1.64) but had borderline ERRAT (75.2) and Verify3D (30.25%). ERRAT and Verify3D are global metrics that are disproportionately influenced by flexible N- and C-terminal regions. For MHQ1860809.1, the AlphaFold2 pLDDT is above 90 across the central Rossmann fold core, with low confidence confined to the N- and C-terminal tails (Fig. 4A). This model was therefore retained because of its high per-residue confidence and its otherwise acceptable global metrics, while its global ERRAT and Verify3D scores are explicitly acknowledged to fall below the applied thresholds. MHQ1859537.1 (PROCHECK 89.4%, QMEAN *Z*-score −4.19) and MHQ1860643.1 (PROCHECK 85.8%, ERRAT 76.98, Verify3D 35.19%) were discarded. Finally, MHQ1860809.1 and MHQ1862734.1 were selected for detailed functional annotation, representing the highest-confidence candidates passing all sequential filters rather than the only hypothetical proteins of potential biological interest. The progressive reduction from 218 to two proteins reflects the stringency of the combined stability, subcellular localisation, and conserved-domain criteria, as summarised in Fig. 1.

### Physicochemical properties of the selected proteins

The amino acid sequences of the two selected hypothetical proteins, MHQ1860809.1 and MHQ1862734.1, were resubmitted to the ExPASy ProtParam tool [15] to calculate the molecular weight, theoretical isoelectric point (pI), instability index, aliphatic index, grand average of hydropathicity (GRAVY), extinction coefficient, and amino acid composition.

### Secondary structure prediction

The secondary structures of MHQ1860809.1 and MHQ1862734.1 were predicted using two complementary computational methods, PSIPRED [28] and SOPMA [29]. PSIPRED employs neural networks and position-specific scoring matrices for high-accuracy predictions, while SOPMA predicts the percentages of α-helices, extended strands, β-turns, and random coils using default parameters. The results obtained from the two methods were compared to ensure consistency.

### Homology identification and phylogenetic analysis

Sequence homologues of MHQ1860809.1 and MHQ1862734.1 were identified by BLASTp searches against the non-redundant (nr) protein database. For each protein, the 10 most significant matches, those with the lowest *E*-values, were retained. Multiple sequence alignment, alignment trimming, and phylogenetic tree inference were performed using the NGPhylogeny.fr one‑click pipeline [30]. The pipeline employs MAFFT for multiple sequence alignment, BMGE for alignment trimming, and PhyML for maximum-likelihood phylogenetic tree construction.

### Active site prediction

Ligand-binding pockets were predicted with PrankWeb [31], using the relaxed AlphaFold2 models of MHQ1860809.1 and MHQ1862734.1 as input. The Default model with conservation prediction mode was selected, which integrates sequence conservation information to enhance pocket ranking. The top-ranked pocket, together with its P2Rank score, probability, number of residues, and average conservation, was recorded for each protein.

### Structural similarity search

The relaxed AlphaFold2 models of MHQ1860809.1 and MHQ1862734.1 were submitted to the Foldseek server [32], to search the Protein Data Bank (PDB) for structurally similar proteins using the PDB100 database and TM-align mode. A TM-score greater than 0.5 was considered indicative of the same overall fold. The top ten annotated hits, together with their TM-scores and sequence identities, were retained for functional inference.

### Enzyme function and Gene Ontology prediction

The amino acid sequences of MHQ1860809.1 and MHQ1862734.1 were submitted to the COFACTOR server [33] for prediction of Enzyme Commission (EC) numbers, Gene Ontology (GO) terms, and ligand-binding residues. The consensus EC numbers and GO annotations with the highest confidence scores (CscoreGO ≥ 0.4) were recorded.

### KEGG pathway annotation

The amino acid sequences of MHQ1860809.1 and MHQ1862734.1 were submitted to the BlastKOALA server [34] for KEGG Orthology (KO) assignment using the reference prokaryotic protein database. The assigned KO numbers and associated pathway identifiers were recorded.

### Molecular docking

To evaluate whether MHQ1860809.1 can accommodate a nucleotide cofactor, molecular docking of NAD^+^ was performed using CB-Dock2 [35]. The relaxed AlphaFold2 model of MHQ1860809.1 was uploaded together with the NAD^+^ structure obtained from PubChem. The server performed blind docking by detecting cavities on the protein surface and docking the ligand into each cavity using AutoDock Vina [36]. The top-ranked docking pose and its binding energy (Vina score) were recorded. Docking results are reported as supportive evidence only; Vina scores are empirical scoring values and are not equivalent to binding free energies.

### Summary of web-based tools and servers

A summary of all web-based tools and servers used in this study, along with their URLs and purposes, is provided in Table 1.

**Table 1:** Web-based tools and servers used in this study

Sl	Server	URL	Purpose
1	NCBI Genome	https://www.ncbi.nlm.nih.gov/	Genome retrieval
2	ExPASy ProtParam	https://web.expasy.org/protparam/	Physicochemical properties
3	CELLO v2.5	http://cello.life.nctu.edu.tw/	Subcellular localisation
4	PSORTb	https://www.psort.org/psortb/	Subcellular localisation
5	SOSUIGramN	http://harrier.nagahama-i-bio.ac.jp/sosui/sosuigramn/	Subcellular localisation
6	PSLpred	https://webs.iiitd.edu.in/raghava/pslpred/	Subcellular localisation
7	NCBI CDD	https://www.ncbi.nlm.nih.gov/Structure/bwrpsb/bwrpsb.cgi	Conserved domain search
8	InterProScan	https://www.ebi.ac.uk/interpro/	Conserved domain search
9	SWISS-MODEL	https://swissmodel.expasy.org/	Homology modelling
10	ColabFold	https://colab.research.google.com/github/sokrypton/ColabFold/	De novo structure prediction
11	SAVES server	https://saves.mbi.ucla.edu/	Model quality assessment
12	QMEANDisCo	https://swissmodel.expasy.org/qmean/	Model quality assessment
13	PSIPRED	http://bioinf.cs.ucl.ac.uk/psipred/	Secondary structure prediction
14	SOPMA	https://npsa-prabi.ibcp.fr/cgi-bin/npsa_automat.pl	Secondary structure prediction
15	BLASTp	https://blast.ncbi.nlm.nih.gov/	Homology search
16	NGPhylogeny.fr	https://ngphylogeny.fr/	Phylogenetic analysis
17	PrankWeb	https://prankweb.cz/	Active site prediction
18	Foldseek	https://search.foldseek.com/	Structural similarity search
19	COFACTOR	https://zhanggroup.org/COFACTOR/	GO and EC number prediction
20	BlastKOALA	https://www.kegg.jp/blastkoala/	KEGG pathway annotation
21	PubChem	https://pubchem.ncbi.nlm.nih.gov/	Ligand structure retrieval
22	CB-Dock2	https://cadd.labshare.cn/cb-dock2/	Molecular docking

## Result

### Analysis of physicochemical properties

The physicochemical properties of MHQ1860809.1 and MHQ1862734.1 are summarised in Table 2.

**Table 2:** Results of the ProtParam analysis of the HPs’ physiochemical characteristics

Description	MHQ1860809.1	MHQ1862734.1
Amino acid number	162	161
Molecular weight	18,433.21	18,055.40
Theoretical pI	5.63	4.72
Ext. coefficient	14,105	4595
Total number of negatively charged residues	19	27
Total number of positively charged residues	14	17
Instability index	39.89	38.49
Aliphatic index	108.89	109.25
Grand average of hydropathicity (GRAVY)	0.083	-0.164

Note: Extinction coefficients are given for the oxidised form (all cysteine pairs assumed to form cystines)

MHQ1860809.1 contains 162 amino acids with a molecular weight of 18,433.21 Da and a theoretical pI of 5.63. The instability index is 39.89 and the GRAVY value is 0.083. The protein contains 19 negatively charged residues (Asp + Glu) and 14 positively charged residues (Arg + Lys). The aliphatic index is 108.89. The extinction coefficient for the oxidised form is 14,105 M⁻^1^ cm⁻^1^; the value for the reduced form is 13,980 M⁻^1^ cm⁻^1^.

MHQ1862734.1 contains 161 amino acids with a molecular weight of 18,055.40 Da and a theoretical pI of 4.72. The instability index is 38.49 and the GRAVY value is −0.164. The protein contains 27 negatively charged residues and 17 positively charged residues. The aliphatic index is 109.25. The extinction coefficient for the oxidised form is 4595 M⁻^1^ cm⁻^1^; the value for the reduced form is 4470 M⁻^1^ cm⁻^1^.

### Subcellular localisation prediction of the selected proteins

Both MHQ1860809.1 and MHQ1862734.1 were consistently predicted to be cytoplasmic proteins by all four subcellular localisation tools. The two sequences lack any recognisable signal peptides or transmembrane helices, which is consistent with an intracellular location.

### Identification of conserved domains

The conserved domains detected in MHQ1860809.1 and MHQ1862734.1 are summarised in Table 3.

**Table 3:** Conserved domains identified in the two selected hypothetical proteins

	**Domain / fold**	**Source**	***E*-value**
**MHQ1860809.1**	NAD(P)-binding Rossmann fold	InterPro (SUPERFAMILY)	3.53 × 10^–5^
	MDR superfamily	NCBI CD Search	5.85 × 10^–3^
**MHQ1862734.1**	ARM repeat (Armadillo type)	InterPro (SUPERFAMILY)	5.76 × 10^–7^

For MHQ1860809.1, InterProScan identified a NAD(P)-binding Rossmann fold domain (SUPERFAMILY SSF51735, *E*-value = 3.53 × 10^–5^; Gene3D G3DSA:3.40.50.720, *E*-value = 2.4 × 10^–6^). NCBI CD-Search detected a multi-drug resistance (MDR) superfamily domain (cl16912, *E*-value = 5.85 × 10^–3^).

For MHQ1862734.1, CD-Search returned no significant domain at the chosen threshold (*E*-value < 0.01). InterProScan revealed an ARM repeat (Armadillo-type fold) (SUPERFAMILY SSF48371, IPR016024, *E*-value = 5.76 × 10^–7^).

All reported domain assignments had *E*-values below 0.01.

### Secondary structure prediction

The secondary structures of MHQ1860809.1 and MHQ1862734.1 were predicted using PSIPRED and SOPMA.

**Fig. 2:**
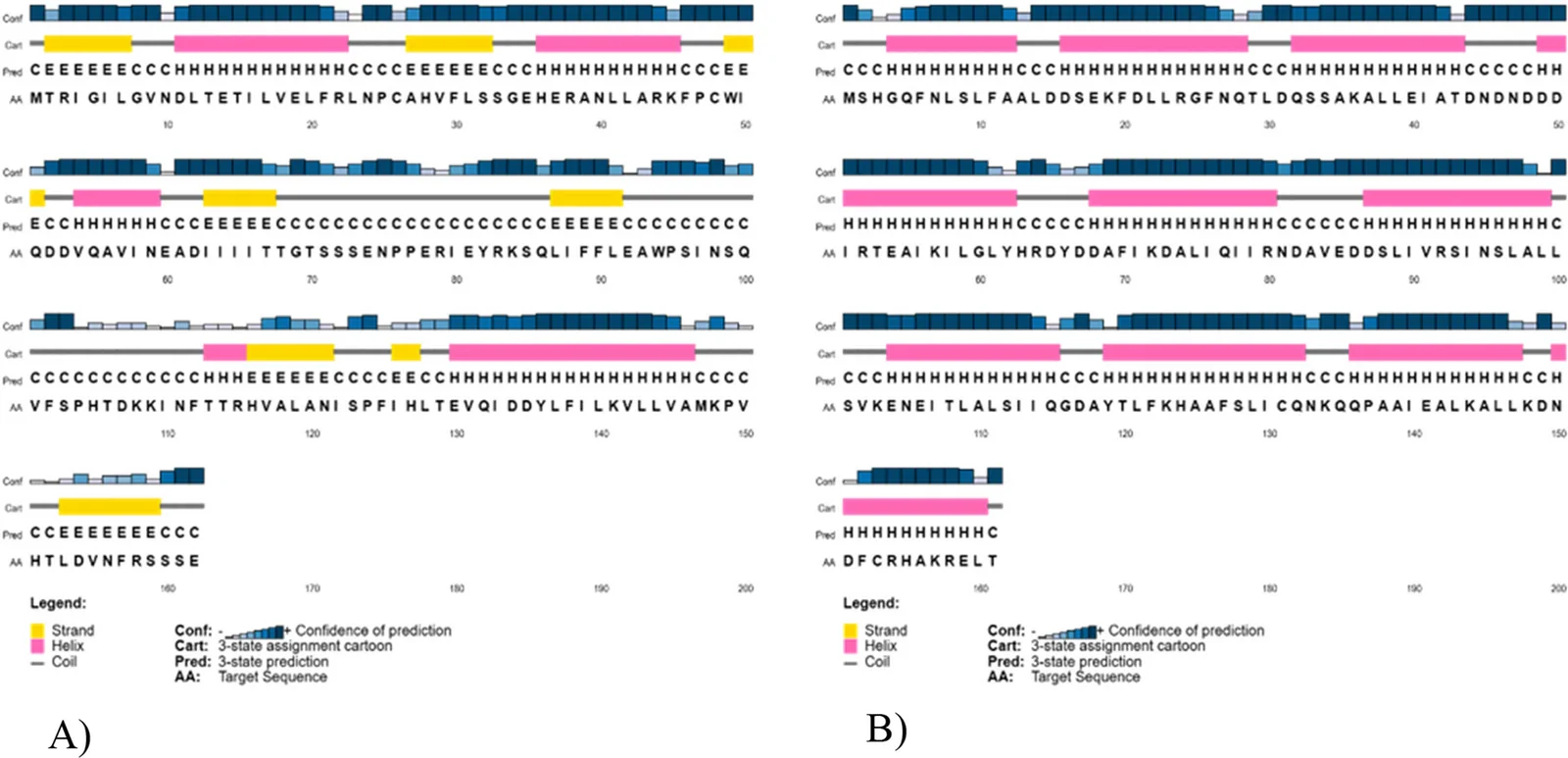
Secondary structure prediction of MHQ1860809.1 and MHQ1862734.1 generated by the PSIPRED server. **A** MHQ1860809.1. **B** MHQ1862734.1. Yellow, pink, and grey represent strand, helix, and coil structures, respectively

For MHQ1860809.1, SOPMA assigned 44.44% of residues as α-helices, 20.37% as extended strands, 3.70% as β-turns, and 31.48% as random coils. PSIPRED assigned 29.6% as helix, 24.7% as strand, and 45.7% as coil (Fig. 2A). For MHQ1862734.1, SOPMA predicted 71.43% α-helices, 1.86% extended strands, 4.35% β-turns, and 22.36% random coils. PSIPRED predicted 76.4% helix, 0.0% strand, and 23.6% coil (Fig. 2B). The secondary structure predictions from PSIPRED and SOPMA showed similar distributions for each protein.

### Homology identification and phylogenetic analysis

For MHQ1860809.1, the 10 most significant BLASTp matches were from *Kosakonia* and closely related *Enterobacteriaceae* (Table 4). The top hits shared 95–100% sequence identity and were mostly annotated as hypothetical proteins. Several hits from *Klebsiella*, *Serratia*, and unclassified *Kosakonia* were annotated as NAD(P)-binding domain-containing protein or NADP oxidoreductase.

**Table 4:** Top ten BLASTp hits for MHQ1860809.1

Rank	Accession	Description	Organism	Identity (%)	*E*-value
1	WP_480161535.1	Hypothetical protein	*Kosakonia cowanii*	100	7.23E-115
2	HCO1310430.1	Hypothetical protein	*Kosakonia cowanii*	98.765	1.18E-113
3	WP_480161459.1	Hypothetical protein	*Kosakonia cowanii*	98.148	1.58E-112
4	WP_289745931.1	Hypothetical protein	*Kosakonia cowanii*	96.914	5.30E-112
5	WP_454125355.1	Hypothetical protein	*Kosakonia cowanii*	96.296	1.70E-111
6	WP_215231192.1	Hypothetical protein	*Kosakonia cowanii*	95.679	1.64E-110
7	WP_261659471.1	Hypothetical protein	*Kosakonia cowanii*	95.062	3.62E-110
8	EPE1850217.1	Hypothetical protein	*Kosakonia cowanii*	96.296	5.09E-110
9	WP_139569374.1	Hypothetical protein	*Kosakonia cowanii*	96.296	1.41E-109
10	WP_319550979.1	Hypothetical protein	*Kosakonia cowanii*	95.679	3.15E-109

For MHQ1862734.1, the closest homologues were found within *Kosakonia cowanii*, with the top hit sharing 100% identity (Table 5). All top 10 hits were annotated as hypothetical proteins. Sequences from *Escherichia*, *Salmonella*, and *Paenibacillus* were annotated as HEAT repeat domain-containing protein.

**Table 5:** Top ten BLASTp hits for MHQ1862734.1

Rank	Accession	Description	Organism	Identity (%)	*E*-value
1	WP_480160633.1	Hypothetical protein	*Kosakonia cowanii*	100	1.82E-109
2	WP_483558511.1	Hypothetical protein	*Kosakonia cowanii*	96.273	2.47E-105
3	WP_277174105.1	Hypothetical protein	*Kosakonia cowanii*	96.273	2.55E-105
4	WP_446427473.1	Hypothetical protein	*Kosakonia cowanii*	96.273	3.35E-105
5	WP_458676393.1	Hypothetical protein	*Kosakonia cowanii*	78.261	4.07E-82
6	WP_433690403.1	Hypothetical protein	*Kosakonia cowanii*	78.261	6.73E-80
7	WP_232045113.1	Hypothetical protein	*Kosakonia cowanii*	78.261	9.69E-80
8	WP_319551328.1	Hypothetical protein	*Kosakonia cowanii*	76.398	1.83E-79
9	WP_305207053.1	Hypothetical protein	*Kosakonia cowanii*	77.019	1.26E-78
10	WP_413311870.1	Hypothetical protein	*Kosakonia cowanii*	76.398	5.52E-78

**Fig. 3:**
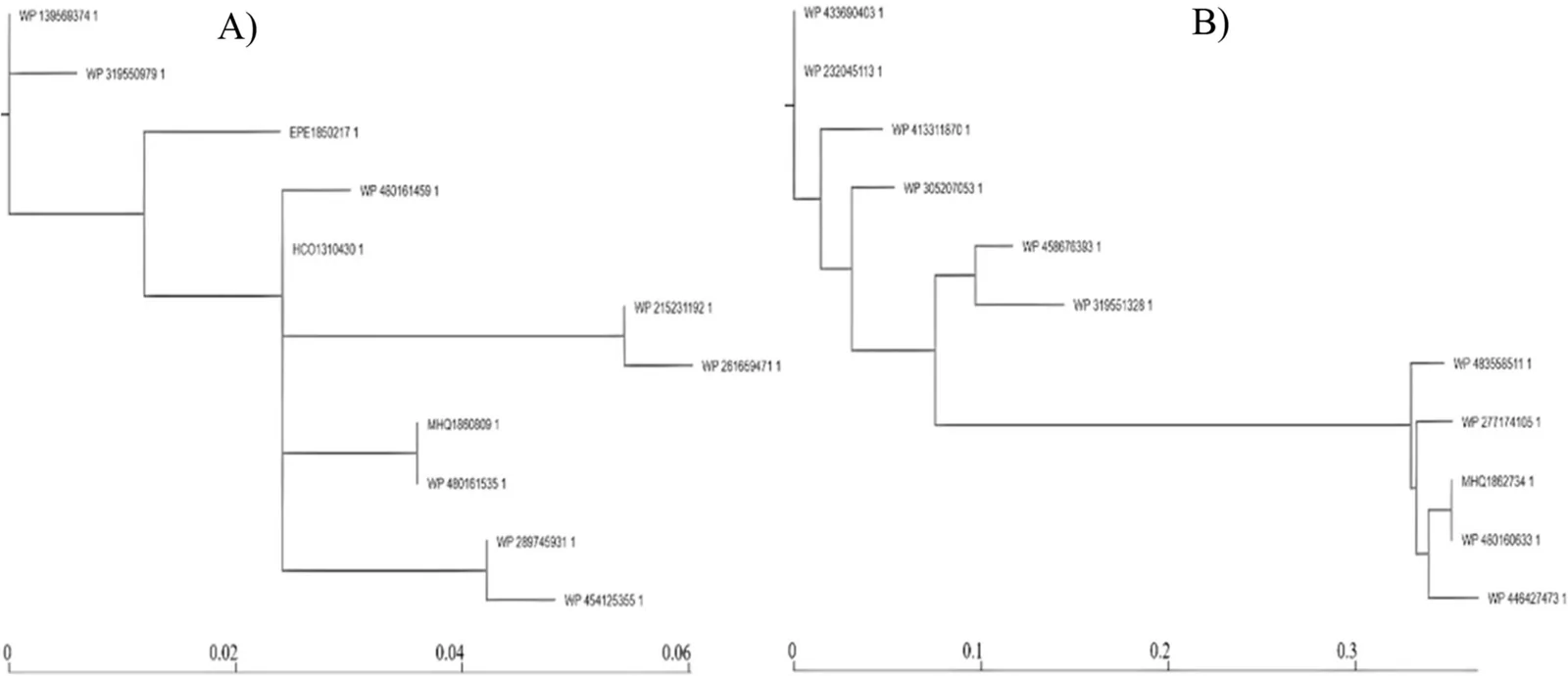
Phylogenetic analysis of the selected hypothetical proteins. **A** Maximum-likelihood tree of MHQ1860809.1 and its top BLASTp hits. **B** Maximum-likelihood tree of MHQ1862734.1 and its top BLASTp hits

The phylogenetic trees constructed from the top 10 homologues of each protein (Fig. 3) show that both MHQ1860809.1 and MHQ1862734.1 cluster tightly with other *Kosakonia* proteins, while also displaying evolutionary links to functionally annotated proteins in other enterobacterial species.

### Three‑dimensional structure prediction and validation

**Fig. 4:**
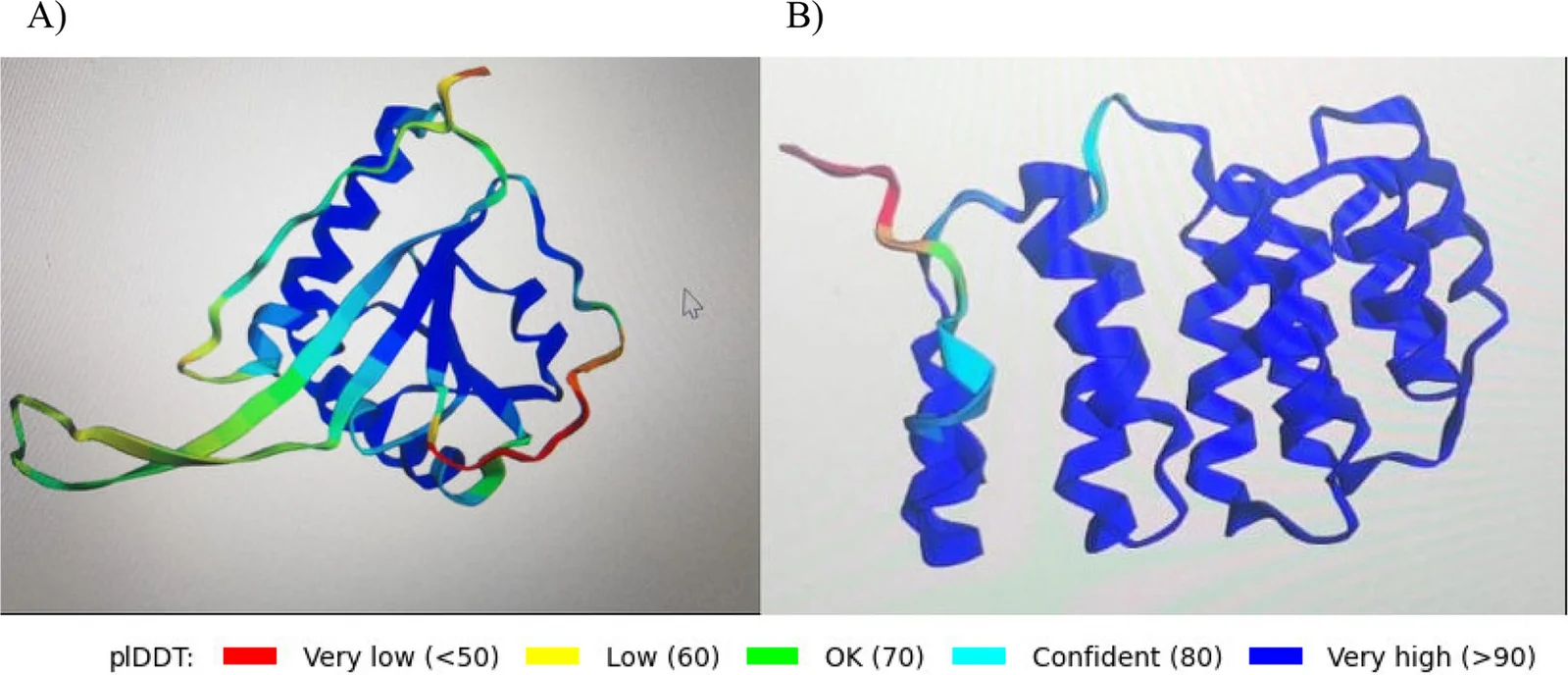
AlphaFold2-predicted three-dimensional structures coloured by pLDDT confidence. **A** MHQ1860809.1. **B** MHQ1862734.1. Blue indicates high confidence (pLDDT > 90) and red indicates low confidence (pLDDT < 50)

The three-dimensional structures of MHQ1860809.1 and MHQ1862734.1 were predicted de novo by AlphaFold2 through the ColabFold environment. The top-ranked relaxed models were retained for each protein. The models are shown as cartoon representations colourised by the predicted local distance difference test (pLDDT), where blue indicates pLDDT > 90 and red indicates pLDDT < 50 (Fig. 4).

For MHQ1860809.1, the model displayed a mixed α/β topology. Most residues in the central region had pLDDT scores above 90, while residues at the N- and C-termini had lower pLDDT scores. For MHQ1862734.1, the model displayed a predominantly α-helical structure. Most residues had pLDDT scores above 90, with lower scores observed only in a short loop region and at the termini.

The geometric and stereochemical quality of both models was assessed using PROCHECK, ERRAT, Verify3D, and QMEANDisCo. The PROCHECK Ramachandran plot showed 92.6% of residues in the most favoured regions for MHQ1860809.1 and 91.6% for MHQ1862734.1. The validation scores are summarised in Table 6. It should be noted that MHQ1860809.1 falls slightly below the ERRAT and Verify3D thresholds at the full-length level; however, its core region shows uniformly high pLDDT confidence (Fig. 4A).

**Table 6:** Validation scores for the two selected hypothetical proteins

Protein	PROCHECK (% most favoured)	ERRAT (Quality Factor)	Verify3D (% residues ≥ 0.1)	QMEAN *Z*‑score
MHQ1860809.1	92.6	75.21	30.25	− 1.64
MHQ1862734.1	91.6	100.0	52.17	− 0.29

### Active site prediction

Potential ligand-binding pockets were predicted using PrankWeb with the relaxed AlphaFold2 models of MHQ1860809.1 and MHQ1862734.1.

For MHQ1860809.1, a single binding pocket was identified (Table 7). The pocket comprises 18 residues, with a P2Rank score of 6.35, a probability of 0.345, and an average evolutionary conservation of 2.296. For MHQ1862734.1, no ligand-binding pocket was detected by PrankWeb.

**Table 7:** PrankWeb ligand‑binding pocket analysis for MHQ1860809.1

Protein	Pocket	Score	Probability	No. of residues	Avg. conservation
MHQ1860809.1	1	6.35	0.345	18	2.296
MHQ1862734.1 is not listed because no binding pocket was identified					

### Structural similarity search

Foldseek structural similarity search against the PDB100 database identified 761 structurally related proteins for MHQ1860809.1 and 242 for MHQ1862734.1. The top ten hits for each protein, ranked by TM-score, are presented in Tables 8 and 9.

**Table 8:** Top ten structural homologues of MHQ1860809.1

Rank	PDB ID	Description	Organism	TM-score	Seq. Id. (%)
1	3gt0	Pyrroline-5-carboxylate reductase	*Bacillus cereus*	0.548	9.0
2	3tri	Pyrroline-5-carboxylate reductase	*Coxiella burnetii*	0.506	7.8
3	5bse	Pyrroline-5-carboxylate reductase	*Medicago truncatula*	0.505	7.4
4	5bsh	Pyrroline-5-carboxylate reductase with L-Proline	*Medicago truncatula*	0.500	6.9
5	3b1f	Prephenate dehydrogenase	*Streptococcus mutans*	0.486	7.0
6	3guc	Ubiquitin-activating enzyme 5 with AMPPNP	*Homo sapiens*	0.523	5.1
7	3dzb	Prephenate dehydrogenase	*Streptococcus thermophilus*	0.488	8.7
8	5uau	Pyrroline-5-carboxylate reductase with proline	*Homo sapiens*	0.485	7.2
9	7mqv	Prephenate dehydrogenase in complex with NAD	*Bacillus anthracis*	0.483	6.4
10	3dzb	Prephenate dehydrogenase	*Streptococcus thermophilus*	0.479	9.2

**Table 9:** Top ten structural homologues of MHQ1862734.1

Rank	PDB ID	Description	Organism	TM-score	Seq. Id. (%)
1	4xri	Importin beta	*Thermochaetoides thermophila*	0.477	4.0
2	7bfq	Integrator cleavage module INTS4	*Homo sapiens*	0.603	6.8
3	6qh7	AP2 clathrin adaptor mu2 core	*Homo sapiens*	0.527	10.6
4	7n6g	C1 central pair protein	*Chlamydomonas reinhardtii*	0.473	6.4
5	5owu	Importin beta Kap95 with Nup1	*Saccharomyces cerevisiae*	0.470	6.0
6	7cun	Integrator-PP2A complex	*Homo sapiens*	0.465	6.8
7	7pks	Integrator complex	*Homo sapiens*	0.464	6.3
8	7cun	Integrator-PP2A complex	*Homo sapiens*	0.462	7.6
9	7z8v	CAND1-SCF-SKP2 complex	*Homo sapiens*	0.443	9.1
10	4nee	AP-2 alpha/sigma2 complex	*Rattus norvegicus*	0.536	6.3

The top 10 structural matches for MHQ1860809.1 are listed in Table 8. The highest TM-score (0.548) was observed with pyrroline-5-carboxylate reductase from *Bacillus cereus* (PDB: 3gt0). The second and third hits were also pyrroline-5-carboxylate reductases from *Coxiella burnetii* (PDB: 3tri, TM-score 0.506) and *Medicago truncatula* (PDB: 5bse, TM-score 0.505). Prephenate dehydrogenases from *Streptococcus mutans* (PDB: 3b1f, TM-score 0.486) and *Streptococcus thermophilus* (PDB: 3dzb, TM-scores 0.488 and 0.479) were also among the top hits. Entry 7mqv (prephenate dehydrogenase from *Bacillus anthracis*) was crystallised in complex with NAD and showed a TM-score of 0.483 with 6.4% sequence identity. The TM-scores for all top 10 hits ranged from 0.48 to 0.55, while sequence identities ranged from 5 to 9%.

The top 10 structural matches for MHQ1862734.1 are listed in Table 9. The highest TM-score (0.603) was observed with the integrator cleavage module INTS4 from *Homo sapiens* (PDB: 7bfq). Importin beta from *Thermochaetoides thermophila* (PDB: 4xri) showed a TM-score of 0.477 with 4.0% sequence identity. AP2 clathrin adaptor mu2 core from *Homo sapiens *(PDB: 6qh7) showed a TM-score of 0.527 with 10.6% sequence identity. Other hits included importin beta Kap95 with Nup1 from *Saccharomyces cerevisiae* (PDB: 5owu, TM-score 0.470), integrator-PP2A complex components (PDB: 7cun, TM-scores 0.465 and 0.462), and the CAND1-SCF-SKP2 complex (PDB: 7z8v, TM-score 0.443). The TM-scores for all top 10 hits ranged from 0.44 to 0.60, while sequence identities ranged from 4 to 11%. No enzymatic domains were observed among the top 10 hits. TM-scores in the range of 0.44–0.60 and sequence identities of 4–11% indicate fold-level similarity only; they do not establish functional conservation.

### Enzyme function and Gene Ontology prediction

The amino acid sequences of MHQ1860809.1 and MHQ1862734.1 were submitted to the COFACTOR server for prediction of Gene Ontology (GO) terms, Enzyme Commission (EC) numbers, and ligand-binding sites based on structural homology. The results for the two proteins are presented separately below.

#### MHQ1860809.1

Gene Ontology predictions: COFACTOR predicted a molecular function of oxidoreductase activity acting on the CH-NH group of donors with NAD or NADP as acceptor (GO:0016646) with a confidence score (CscoreGO) of 0.58. For biological process, the protein was predicted to be involved in proline metabolic process (GO:0006560, CscoreGO 0.58), glutamine family amino acid biosynthetic process (GO:0009084, CscoreGO 0.58), and carboxylic acid biosynthetic process (GO:0046394, CscoreGO 0.58). For cellular component, the protein was predicted to localise to the cytoplasm (GO:0005737, CscoreGO 0.73). Additional predicted cellular components included intracellular part (GO:0044424, CscoreGO 0.93) and cytoplasmic part (GO:0044444, CscoreGO 0.60). The complete set of confident GO predictions is summarised in Table 10.

**Table 10:** Predicted Gene Ontology terms for MHQ1860809.1

Category	GO ID	Term	CscoreGO
Molecular Function	GO:0016646	Oxidoreductase activity, acting on the CH-NH group of donors, NAD or NADP as acceptor	0.58
Biological Process	GO:0006560	Proline metabolic process	0.58
Biological Process	GO:0009084	Glutamine family amino acid biosynthetic process	0.58
Biological Process	GO:0046394	Carboxylic acid biosynthetic process	0.58
Cellular Component	GO:0005737	Cytoplasm	0.73
Cellular Component	GO:0044424	Intracellular part	0.93
Cellular Component	GO:0044444	Cytoplasmic part	0.60

Enzyme Commission number prediction: No confident Enzyme Commission (EC) number could be assigned to MHQ1860809.1. The top five EC predictions from structural homologues all had very low confidence scores (CscoreEC ≤ 0.060). The sequence identities to experimentally characterised enzymes ranged from 5 to 9% in the Foldseek structural similarity search.

Ligand-binding site prediction: COFACTOR identified a putative NAD(P)-binding pocket in MHQ1860809.1. The top-ranked ligand-binding site template was PDB entry 2ahrA (chain A), a pyrroline-5-carboxylate reductase crystallised with NADP^+^, with a binding site similarity score (BS-score) of 0.60. The predicted binding site residues include positions 8, 9, 10, 11, 12, 54, 67, 68, 69, 91, and 92.

#### MHQ1862734.1

Gene Ontology predictions: COFACTOR could not confidently predict any molecular function or biological process terms. All predicted terms in these categories had CscoreGO values between 0.14 and 0.14 (below the 0.4 confidence threshold).

For cellular component, COFACTOR predicted cytoplasmic localisation (GO:0005737, CscoreGO 0.50) and cytosolic localisation (GO:0005829, CscoreGO 0.67). Additional predicted cellular components included intracellular part (GO:0044424, CscoreGO 0.92), intracellular membrane-bounded organelle (GO:0043231, CscoreGO 0.83), intracellular organelle part (GO:0044446, CscoreGO 0.75), cytoplasmic part (GO:0044444, CscoreGO 0.75), protein complex (GO:0043234, CscoreGO 0.58), and cell part (GO:0044464, CscoreGO 1.00). The protein was also predicted to localise to the nucleus (GO:0005634, CscoreGO 0.67) and nuclear part (GO:0044428, CscoreGO 0.50). The confident cellular component predictions are summarised in Table 11.

**Table 11:** Predicted Gene Ontology cellular component terms for MHQ1862734.1

GO ID	Term	CscoreGO
GO:0044464	Cell part	1.00
GO:0044424	Intracellular part	0.92
GO:0043231	Intracellular membrane-bounded organelle	0.83
GO:0044446	Intracellular organelle part	0.75
GO:0044444	Cytoplasmic part	0.75
GO:0005829	Cytosol	0.67
GO:0005634	Nucleus	0.67
GO:0043234	Protein complex	0.58
GO:0044428	Nuclear part	0.50
GO:0016020	Membrane	0.50
GO:0005737	Cytoplasm	0.50

Enzyme Commission number prediction: No confident Enzyme Commission (EC) number could be assigned to MHQ1862734.1. The top five EC predictions all had very low confidence scores (CscoreEC = 0.060).

Ligand-binding site prediction: COFACTOR did not identify any significant ligand-binding pocket in MHQ1862734.1. The top-ranked ligand-binding site predictions had very low confidence scores (CscoreLB = 0.02) and binding site similarity scores below 0.5 (BS-score ≤ 0.49).

### KEGG pathway annotation

The amino acid sequences of MHQ1860809.1 and MHQ1862734.1 were submitted to the BlastKOALA server for KEGG Orthology (KO) assignment using the reference prokaryotic protein database. No significant KEGG Orthology (KO) number could be assigned to either MHQ1860809.1 or MHQ1862734.1.

### Molecular docking of NAD^+^

Molecular docking of NAD^+^ into the relaxed AlphaFold2 model of MHQ1860809.1 was performed using CB-Dock2. Five potential binding cavities were detected. The docking results for all five cavities are summarised in Table 12.

**Table 12:** Molecular docking results of NAD⁺ with MHQ1860809.1

Cavity ID	Vina Score (kcal/mol)	Volume (Å^3^)	Center (x, y, z)
C2	−7.8	501	8, −7, −8
C1	−6.9	3978	7, 9, 0
C4	−6.7	37	7, 5, 12
C5	−6.6	36	−12, 7, −13
C3	−6.5	91	−12, 2, −3

The top-ranked cavity (C2) yielded a Vina score of −7.8 kcal/mol with a cavity volume of 501 Å^3^. The interacting residues in Cavity C2 include LEU:7, GLY:8, VAL:9, ASN:10, ASP:11, LEU:12, SER:32, SER:33, GLY:34, GLU:35, HIS:36, GLU:37, ARG:38, ALA:39, VAL:54, ILE:66, THR:67, THR:68, GLY:69, THR:70, SER:71, SER:72, SER:73, GLU:74, ASN:75, PRO:76, PRO:77, ILE:80, TYR:82, PHE:90, LEU:91, GLU:92, ALA:93, and TRP:94.

The second-ranked cavity (C1) produced a Vina score of −6.9 kcal/mol with a cavity volume of 3978 Å^3^. Cavities C4, C5, and C3 produced Vina scores of −6.7, −6.6, and −6.5 kcal/mol, respectively. The docking result should be interpreted as consistent with, but not proof of, NAD⁺ binding. Docking of NAD⁺ into a Rossmann-fold protein is expected given the fold's canonical cofactor-binding role, and Vina scores in the range observed here are routine for medium-sized pockets. No negative control, molecular dynamics simulation, or MM/PBSA calculation was performed in this study; such analyses would be required to strengthen the docking evidence and are proposed as future work.

## Discussion

The goal of this study was to generate testable functional hypotheses for hypothetical proteins in *K. cowanii* F52 using a systematic computational pipeline. From 218 initial candidates, we identified two proteins: MHQ1860809.1, a putative NAD(P)-dependent oxidoreductase with a canonical Rossmann fold potentially involved in proline biosynthesis, and MHQ1862734.1, which adopts an ARM-repeat fold of unknown function. These findings provide a computational basis for further experimental investigation of the salt-tolerant, plant-growth-promoting activity of *K. cowanii* F52.

### MHQ1860809.1 as an NADP‑binding Rossmann‑fold protein

The AlphaFold2 model of MHQ1860809.1 is consistent with a canonical Rossmann fold, a central parallel β-sheet flanked by α-helices, which is the classic scaffold for binding NAD(P) cofactors [37, 38]. Conserved domain searches confirmed a NAD(P)-binding Rossmann fold domain (InterPro SSF51735, *E*-value = 3.53 × 10⁻^5^). Molecular docking of NAD⁺ into the predicted binding cavity (volume 501 Å^3^) gave a Vina score of −7.8 kcal/mol, and the interacting residues (Gly8, Val9, Asn10, Asp11, Leu12, Thr67-70) match the Rossmann motif that anchors the adenine ring. The structural data are consistent with an NAD(P)-dependent enzymatic function, but the low sequence identity and modest TM-scores mean that this assignment remains a hypothesis rather than a demonstration.

Foldseek structural similarity searches placed MHQ1860809.1 with pyrroline-5-carboxylate reductase (P5CR; TM-score 0.548, sequence identity only 9%). P5CR catalyses the final step of proline biosynthesis: the NADPH-dependent reduction of Δ^1^‑pyrroline‑5‑carboxylate to proline [39, 40]. Proline is a well-established osmoprotectant that accumulates in both bacteria and plants under salt stress [41]. Many PGPR upregulate proline synthesis to maintain osmotic balance [42], and proline secreted into the rhizosphere can be taken up by plant roots, potentially enhancing host stress tolerance [43, 44]. GO annotation further predicted oxidoreductase activity (GO:0016646) and proline metabolic process (GO:0006560). MHQ1860809.1 is therefore structurally consistent with a P5CR-like Rossmann fold enzyme. This remains a computational hypothesis requiring experimental confirmation; it does not establish that the protein produces proline or contributes to salt tolerance in *K. cowanii* F52. Canonical P5CRs are approximately 300 amino acids and dimeric, whereas MHQ1860809.1 is only 162 amino acids. However, the catalytic Rossmann fold domain of P5CR is itself approximately 150–170 amino acids, suggesting that MHQ1860809.1 corresponds to the catalytic core rather than the full-length enzyme. Definitive assignment of P5CR activity would require experimental characterisation. A multiple sequence alignment with characterised P5CRs to confirm conservation of catalytic residues, together with more detailed docking and free-energy estimates, would substantially strengthen this hypothesis and is proposed as future work.

### MHQ1862734.1 as an ARM-repeat fold with no catalytic cavity

The AlphaFold2 model of MHQ1862734.1 reveals an all-α-helical superhelix, the signature of ARM (Armadillo) repeats [45, 46]. InterProScan confirmed an ARM repeat domain (SSF48371, *E* = 5.76 × 10⁻⁷). Unlike the Rossmann fold protein, no ligand‑binding cavity was detected by PrankWeb or COFACTOR, and no Enzyme Commission (EC) number could be assigned. Both observations are consistent with a non‑catalytic, scaffolding role. Foldseek structural similarity searches placed MHQ1862734.1 with importin‑β (TM‑score 0.477), AP‑2 adaptor core (TM‑score 0.527), and integrator complex INTS4 (TM‑score 0.603). All are canonical scaffolds that organise multiprotein complexes without enzymatic activity [47, 48]. These structural matches indicate that MHQ1862734.1 adopts an ARM/HEAT-type fold; functional inference from cross-kingdom structural matches is not warranted.

ARM repeats are protein‑protein interaction platforms that can bind multiple partners simultaneously, enabling them to act as hubs in signalling networks [49, 50]. In bacteria, ARM-repeat proteins have been implicated in stress sensing. For instance, *Bacillus subtilis* YjbH uses an ARM‑repeat region to regulate the general stress response [51], and the Xanthomonas effector XopL employs ARM repeats to manipulate host immunity [52]. However, no interaction partners of MHQ1862734.1 were identified in this study, and no evidence links this protein to salinity sensing or to the proline biosynthesis pathway.

### Overall summary

The two hypothetical proteins identified in this study are structurally distinct. MHQ1860809.1 is predicted to adopt a Rossmann fold consistent with an NAD(P)-dependent oxidoreductase, while MHQ1862734.1 adopts an ARM/HEAT-type fold. Although both proteins are predicted to be cytoplasmic, no evidence presented here demonstrates that they function together, and we do not propose a functional or regulatory relationship between them. Direct interaction between the two HPs has not been experimentally demonstrated. Their co-occurrence in the genome does not by itself imply functional coupling.

Most PGPR studies rely on sequence‑based homology (e.g. BLAST), which fails for proteins with low sequence identity [7]. Our sequence identities to annotated proteins were only 4–11%, far below the reliable threshold. Structure-based annotation of hypothetical proteins is an established strategy rather than a novel methodology; the contribution of this work therefore lies in the specific, testable hypotheses generated for *Kosakonia cowanii* F52, a genome for which no curated functional database is available.

All functional assignments in this study are computational and require experimental validation. The low sequence identities (4–11%) between our hypothetical proteins and their structural homologues mean that functional inference relies on structural conservation, which is robust but not absolute. Molecular docking suggests NAD⁺ binding to MHQ1860809.1 but does not prove enzymatic activity or substrate specificity; no negative control, molecular dynamics simulation, or MM/PBSA analysis was performed, and these are proposed as future work. For MHQ1862734.1, specific interaction partners have not been identified, and its function remains unknown. No genetic knockout or plant inoculation experiments were performed, so the physiological relevance of both proteins for salt tolerance and plant growth promotion has not been directly tested. In addition, only two of the 218 hypothetical proteins were characterised in detail, and comparative genomic analyses were not performed. The absence of experimental validation is the principal limitation of this study; the annotations presented here are computational predictions and should be treated as hypotheses. Nevertheless, hypothesis-generating annotation studies of understudied genomes are a useful contribution, particularly when no prior functional data exist and the predictions are directly testable.

Experimental validation should begin with targeted knockout mutants of the genes encoding MHQ1860809.1 and MHQ1862734.1 in *K. cowanii* F52. The MHQ1860809.1 strain would test proline accumulation and growth under high salinity, while the MHQ1862734.1 strain would test its physiological role under high salinity. Genome-wide application of this approach to all 218 hypothetical proteins, together with comparative analyses across *Kosakonia* genomes, will be required to determine whether the two proteins studied here are representative of a broader pattern. Yeast two-hybrid or co-immunoprecipitation could identify interaction partners of MHQ1862734.1. Finally, plant inoculation assays under saline conditions would confirm the physiological relevance of both proteins for plant growth promotion.

## Conclusion

We have generated experimentally testable hypotheses for two hypothetical proteins in *Kosakonia cowanii* F52: MHQ1860809.1 is structurally consistent with a putative NAD(P)-dependent oxidoreductase potentially involved in proline biosynthesis, whereas MHQ1862734.1 adopts an ARM-repeat fold of unknown function. These findings provide a basis for future experimental investigation of these two proteins and their potential roles in *K. cowanii* biology. The approach employed here may be adapted for hypothesis generation in other understudied plant-associated bacteria. All functional predictions presented here are computational hypotheses and require experimental validation.

## Data Availability

The genome sequence of *Kosakonia cowanii* strain F52 used in this study is publicly available from the NCBI nucleotide database under accession number CM160069.1. All structural models, docking results generated during this study are available from the corresponding author upon reasonable request.
